# The nasopharyngeal microbiota of beef cattle before and after transport to a feedlot

**DOI:** 10.1186/s12866-017-0978-6

**Published:** 2017-03-22

**Authors:** Devin B. Holman, Edouard Timsit, Samat Amat, D. Wade Abbott, Andre G. Buret, Trevor W. Alexander

**Affiliations:** 1 United States Department of Agriculture, National Animal Disease Center, Agricultural Research Service, Ames, IA USA; 2 Department of Production Animal Health, University of Calgary, Calgary, AB Canada; 3 Lethbridge Research Centre, Agriculture and Agri-Food Canada, 5403 1st Avenue South, Lethbridge, AB T1J 4B1 Canada; 4 Department of Biological Sciences, University of Calgary, Calgary, AB Canada

**Keywords:** Cattle, Nasopharyngeal microbiota, Microbiome, Feedlot, Bovine respiratory disease, 16S rRNA gene, Livestock

## Abstract

**Background:**

The nasopharyngeal (NP) microbiota plays an important role in bovine health, comprising a rich and diverse microbial community. The nasopharynx is also the niche for potentially pathogenic agents which are associated with bovine respiratory disease (BRD), a serious and costly illness in feedlot cattle. We used 14 beef heifers from a closed and disease-free herd to assess the dynamics of the NP microbiota of cattle that are transported to a feedlot. Cattle were sampled prior to transport to the feedlot (day 0) and at days 2, 7, and 14.

**Results:**

The structure of the NP microbiota changed significantly over the course of the study, with the largest shift occurring between day 0 (prior to transport) and day 2 (P < 0.001). Phylogenetic diversity and richness increased following feedlot placement (day 2; P < 0.05). The genera *Pasteurella, Bacillus*, and *Proteus* were enriched at day 0, *Streptococcus* and *Acinetobacter* at day 2, *Bifidobacterium* at day 7, and *Mycoplasma* at day 14. The functional potential of the NP microbiota was assessed using PICRUSt, revealing that replication and repair, as well as translation pathways, were more relatively abundant in day 14 samples. These differences were driven mostly by *Mycoplasma*. Although eight cattle were culture-positive for the BRD-associated bacterium *Pasteurella multocida* at one or more sampling times, none were culture-positive for *Mannheimia haemolytica* or *Histophilus somni*.

**Conclusions:**

This study investigated the effect that feedlot placement has on the NP microbiota of beef cattle over a 14-d period. Within two days of transport to the feedlot, the NP microbiota changed significantly, increasing in both phylogenetic diversity and richness. These results demonstrate that there is an abrupt shift in the NP microbiota of cattle after transportation to a feedlot. This may have importance for understanding why cattle are most susceptible to BRD after feedlot placement.

**Electronic supplementary material:**

The online version of this article (doi:10.1186/s12866-017-0978-6) contains supplementary material, which is available to authorized users.

## Background

The nasopharyngeal (NP) microbiota of cattle is comprised of a wide range of bacteria, the proportions of which may vary between individual animals and throughout placement in feedlots [1–3]. Colonization of the nasopharynx begins shortly after birth and the microbiota continues to change as cattle age [4], although it is typically dominated by members of the Firmicutes, Proteobacteria, and Tenericutes phyla [1–3]. The relatively abundant genera in the NP microbiota include *Corynebacterium*, *Moraxella*, *Mycoplasma*, *Pasteurella*, *Psychrobacter*, and *Staphylococcus* [1, 3, 5]. Until recently, research regarding the bovine respiratory tract has been focused largely on pathogens. However, with an increasing number of studies showing the importance of the host’s microbiota in relation to health [6], it is important to also evaluate the impact of production practices on the microbiota of livestock. In North America, the majority of beef cattle are finished in feedlots after being purchased from auction markets or directly from farms. The majority of bovine respiratory (BRD) cases also occur within the first weeks of feedlot placement. Therefore, determining how the NP microbiota of cattle responds to introduction to the feedlot environment is important for understanding why cattle are most susceptible to BRD during this period.

Bovine respiratory disease is the most significant health problem in feedlot cattle, resulting in considerable economic losses due to mortalities, cost of treatment, reduced feed efficiency, and lower carcass quality [7]. A number of stressors have been associated with an increased risk of developing BRD, including co-infection with viruses, climate, dust, age, commingling of animals from different sources, and transport [8]. The major bacteria associated with BRD include *Mannheimia haemolytica*, *Pasteurella multocida*, *Histophilus somni*, and *Mycoplasma bovis* [9]. These bacterial species are often considered opportunistic pathogens in cattle but their presence in the bovine nasopharynx is also associated with an increased incidence of BRD. For example, cattle positive for *M. haemolytica* at feedlot arrival are more likely to become ill within 10 days [10].

In North America, high risk cattle (e.g. non-vaccinated, low weight, unknown history, presence of BRD in cohort) frequently receive metaphylactic antimicrobials upon arrival to the feedlot to mitigate BRD and reduce colonization by bacterial pathogens [11]. It has previously been reported that 39.2% of high risk cattle receive an injectable antimicrobial following feedlot placement [7]. However, there are serious concerns regarding agricultural antimicrobial use and resistance in animal and human bacterial pathogens [12]. Consequently, new strategies through management or antimicrobial alternatives are required to reduce antimicrobial use in livestock, including the manipulation and supplementation of the various livestock microbiota.

In the present study, we used 16S rRNA gene high-throughput sequencing to characterize the NP microbiota of beef cattle heifers that were transported to a feedlot from a closed herd that was regularly tested to be free of contagious diseases. We used a disease-free herd because the calves all had a similar genetic background, and were not exposed to potential perturbations such as antimicrobial and vaccine administration. We also monitored the cattle for the presence of the BRD-associated bacteria *M. haemolytica*, *P. multocida*, and *H. somni* using culture-based methods. It was hypothesized, based on previous research [1, 3], that cattle would be exposed to novel microorganisms in the feedlot environment resulting in significant changes in their NP microbiota over a 14-d period.

## Methods

### Animal husbandry, experimental design, and sampling of cattle

Fourteen Angus × Herford heifers, approximately 8 months of age with an initial body weight of 290 ± 25 kg, were sourced from a research farm that was established in 1984 and free of the following pathogens: the herd was tested annually for bovine viral diarrhea virus, bovine herpes virus-1, *Leptospira* (serovars Canicola, Pomona, Hardjo, Grippotyphosa, and Copenhageni), *Anaplasma phagocytophilum*, bluetongue virus, and *Brucella abortus*, biannually for *Mycobacterium avium* subspecies *paratuberculosis* and bovine leukosis virus, and every five years for *Mycobacterium bovis*. Cattle positive for any of the above disease agents were removed from the herd. None of the cattle used were administered antimicrobials or vaccines prior to or during the study. Calves were weaned 41 d prior to study enrollment (day −41) and were bunk-fed an alfalfa-barley silage mixed diet in pens. On day 0, calves were transported to the feedlot (distance of 20 km).

Upon arrival at the feedlot, the heifers were not mixed with cattle from other sources and were fed alfalfa-barley silage mixed diets similar to the ones at the disease-free farm. Nasopharyngeal samples were collected from each calf in the study on days 0 (at the disease-free farm prior to shipment), 2, 7, and 14 according to Timsit et al. [3]. Prior to sampling, the nostril was wiped clean with 70% ethanol. Extended guarded swabs (27 cm) with a rayon bud (MW 124, Medical Wire & Equipment, Corsham, England) were used for sampling (Additional file 1: Fig. S1) and swabs were transported to the lab on ice for processing, within one hour of collection. Animals used in this study were cared for according to the guidelines set by the Canadian Council on Animal Care [13] and all experimental procedures involving cattle were approved by the Animal Care Committee of the Lethbridge Research Centre.

### Isolation and detection of bovine respiratory pathogens

Nasopharyngeal swabs were suspended in 1.2 mL of brain heart infusion (BHI) broth with 20% glycerol and vortexed. For isolation of *M. haemolytica* and *P. multocida,* a 100 μl aliquot of the swab suspension was plated onto tryptic soy agar (TSA) plates containing 5% sheep blood, supplemented with 15 μg bacitracin ml^−1^ (Dalynn Biologicals, Inc., Calgary, AB, Canada), and incubated overnight at 37 °C. For culturing of *H. somni*, a 100 μl aliquot of the swab suspension was plated onto TSA plates containing 5% sheep blood without the bacitracin supplement and incubated for 48 h in a 10% CO_2_-enriched environment at 37 °C. Colonies displaying morphology of *M. haemolytica* (white-grey, round, medium-sized, non-mucoid, exhibiting β-haemolysis), *P. multocida* (translucent, greyish in colour, and mucoid in consistency) and *H. somni* (yellowish hue, haemolytic) were confirmed through polymerase chain reaction (PCR) analysis using HotStarTaq Plus Master Mix (Qiagen Canada Inc., Toronto, ON) according to manufacturer’s specifications with primers and annealing conditions described in Table S1 (Additional file 2). Colonies were lysed in Tris–ethylenediaminetetraacetic acid (EDTA) buffer (10 mM Tris–HCl, pH 7.4, 1 mM EDTA, pH 8.0) at 95 °C for 5 min and used as deoxyribonucleic acid (DNA) template (2 μl) in PCR. The swabs were placed in the remaining swab suspension and stored at −80 °C until DNA extraction.

### DNA extraction, PCR amplification, and sequencing of the 16S rRNA gene

Total DNA was extracted from NP swabs using a Qiagen DNeasy Tissue kit (Qiagen Inc., Mississauga, ON, Canada) as previously described [1]. 16S rRNA gene sequence libraries were generated using a two-step PCR protocol. The first PCR step amplified the V4 region of the 16S rRNA gene using the universal bacterial and archaeal primers 515-F (GTGCCAGCMGCCGCGGTAA) and 806-R (GGACTACVSGGGTATCTAAT) [14]. The second PCR step was used to add a unique 10-bp barcode at the 5’ end of each amplicon as well as to add Illumina (Illumina, San Diego, CA, USA) adapter sequences. All PCR amplification and sequencing steps were carried out at Genome Quebec (Montreal, QC, Canada). The 16S rRNA gene amplicons were quantified using a Quant-iT PicoGreen dsDNA assay kit (Invitrogen, Burlington, ON, Canada), pooled in equimolar ratios, and then purified with AMPure XP beads (Beckman Coulter, Mississauga, ON, Canada). Sequencing of 16S rRNA gene amplicons was carried out using an Illumina MiSeq (2 × 250) and the MiSeq Reagent Kit v2 (500 cycles; Illumina) according to manufacturer’s instructions.

### Analysis of 16S rRNA gene sequences

The 16S rRNA gene sequences were processed and analyzed within the QIIME (quantitative insights into microbial ecology) software package v. 1.9.1 [15]. Paired-end reads were joined using fastq-join with a percent allowed maximum difference of 15% and a minimum overlap of 35 bp [16]. Joined sequences were quality filtered with sequences being truncated following three consecutive base calls of a Phred score of less than 25. Sequences were retained only when 75% or more of the original sequence remained after truncation. Chimeric sequences were removed using the UCHIME algorithm [17] implemented in USEARCH v. 6.1544 [18]. Sequences were then assigned to operational taxonomic units (OTUs) at 97% similarity using an open-reference OTU picking method and the SILVA database v. 123 [19]. In this method, sequences that were less than 97% similar to those in the SILVA database were clustered into OTUs using the *de novo* approach and USEARCH. Taxonomy was assigned using the UCLUST consensus taxonomy assigner with a minimum similarity of 0.8 and max accepts of three [18]. Sequences were then aligned using PyNAST [20] and a phylogenetic tree was created using FastTree [21]. OTUs containing fewer than ten sequences were removed prior to analysis, as were sequences classified as mitochondria or chloroplasts.

To account for uneven sequencing depth across samples, each sample was randomly subsampled to 24,500 sequences, with the loss of one sample in the day 0 group. The bacterial and archaeal diversity, evenness, and richness in each sample was calculated within QIIME using the Shannon index [22], phylogenetic diversity (PD whole tree) [23], and equitability. The bacterial and archaeal community structure (beta-diversity) of each group and sampling time was evaluated using the unweighted and weighted UniFrac distances [24] and visualized as principal coordinate analysis (PCoA) plots using Emperor [25].

### Functional analysis of the NP microbiome

The predicted functional analysis of the NP microbiome was performed using PICRUSt (Phylogenetic Investigation of Communities by Reconstruction of Unobserved States) v. 1.0.0. PICRUSt uses the phylogenetic composition of a sample based on 16S rRNA gene sequencing to infer the functional content of the microbiota [26]. For this analysis, sequences were clustered into OTUs in QIIME using the closed reference OTU picking algorithm and the Greengenes 13_5 database [27] and all samples were randomly subsampled to 17,700 sequences. The OTU table was then normalized using the known 16S rRNA gene copy numbers for each representative sequence. The metagenome of each sample was inferred using the KEGG (Kyoto Encyclopedia of Genes and Genomes) Orthology database [28] and functional predictions were made for each sample. The KEGG orthologies (KOs) were categorized into KEGG level 2 pathways with the removal of the non-microbial pathways: organismal systems and human disease. The nearest sequenced taxon index (NSTI) was used to provide a measure of the availability of sequenced reference genomes for each OTU in a sample. It calculates the average branch length for each sample between an OTU and the nearest sequenced reference genome based on the Greengenes reference phylogeny, accounting for OTU abundance [26].

### Statistical analysis

The within-sample or alpha-diversity metrics were compared by sampling time using the PROC MIXED procedure in SAS 9.4 [29]﻿ with sampling time as a repeated measure and individual animal as the random effect. Tukey’s honestly significant difference post-hoc test was then used to compare means and adjust for multiple pairwise comparisons. Linear discriminant analysis effect size (LEfSe) was used to determine the genera that were associated with a specific sampling time. LEfSe uses the Kruskal-Wallis test to identify significantly different (*P* < 0.05) genera among groups of samples and estimates the effect size of each of these using linear discriminant analysis [30]. A linear discriminant analysis (LDA) score of 4.0 was used as the threshold for plotting differentially abundant taxa. The unweighted and weighted UniFrac distances were compared using ANOSIM (analysis of similarities) with 999 permutations. An ANOVA followed by the Benjamini-Hochberg false discovery rate (FDR) correction for multiple comparisons was used to compare the relative abundance of KEGG level 2 pathways by sampling time using STAMP v. 2.1.3 [31]. Bray-Curtis distances [32] were utilized to assess changes in the predicted functional profile of each sample based on KOs. All results were considered significant at *P* < 0.05 or FDR < 0.05.

All 16S rRNA gene sequences were submitted to the NCBI Sequence Read Archive (SRA) under bioproject accession PRJNA296393 (http://www.ncbi.nlm.nih.gov/sra).

## Results

### Isolation and detection of BRD-associated bacteria

Nasopharyngeal swabs were first screened for the BRD-associated bacteria *H. somni*, *M. haemolytica*, and *P. multocida*, using culture-based methods and isolates were confirmed using PCR. Neither *H. somni* nor *M. haemolytica* were isolated during the study period (Additional file 3: Table S2). Interestingly, although *P. multocida* was isolated from the nasopharynx of eight heifers, none of these animals remained positive for *P. multocida* at all four sampling times. Furthermore, by day 14, *P. multocida* was isolated from only one animal.

To compare the culture results with those of the 16S rRNA gene sequencing, the representative sequence for each OTU that was assigned to the genus *Pasteurella* or *Mannheimia* by the UCLUST consensus taxonomy assigner, was further characterized using BLASTn (blast.ncbi.nlm.nih.gov; Additional file 4: Table S3) across all time points. *H. somni* was excluded as it was not detected by culturing or sequencing. *Pasteurella* spp. OTUs comprised 3.2% of total sequences and overall, 28 out of 29 of these OTUs were identified as *P. multocida* based on the top BLASTn results. There were 28 OTUs that were classified as *Mannheimia* spp., with a total relative abundance of 1.5%. However, the majority of these OTUs (19/28) were identified via BLASTn as *M. varigena*, rather than *M. haemolytica* (8/28), which may explain the absence of detection via culturing. Although we did not attempt to culture *Mycoplasma*, due to its potential role in BRD and high prevalence in the 16S rRNA gene sequencing results (13.1% relative abundance), we used BLASTn to identify the five most relatively abundant *Mycoplasma* OTUs. These five OTUs contained more than 99% of *Mycoplasma* sequences and among these sequences, *Mycoplasma bovirhinis* and *Mycoplasma dispar* comprised equal proportions (6.2% each). Candidatus *Mycoplasma haemobos*, with a relative abundance of 0.91% was the only other *Mycoplasma* species among the top five OTUs analyzed.

### Bovine nasopharyngeal microbiota

A total of 3,868,199 archaeal and bacterial 16S rRNA gene sequences, with an average length of 260 bp, remained after primer removal, quality-filtering, and chimera-checking. These sequences were clustered into 6,381 OTUs following the removal of OTUs containing less than ten sequences and prior to random subsampling of each sample to 24,500 sequences. These OTUs were assigned to 28 different phyla and 478 genera. Six phyla, however, accounted for 97% of the sequences: Firmicutes, Proteobacteria, Tenericutes, Actinobacteria, Bacteroidetes, and

**Fig. 1:**
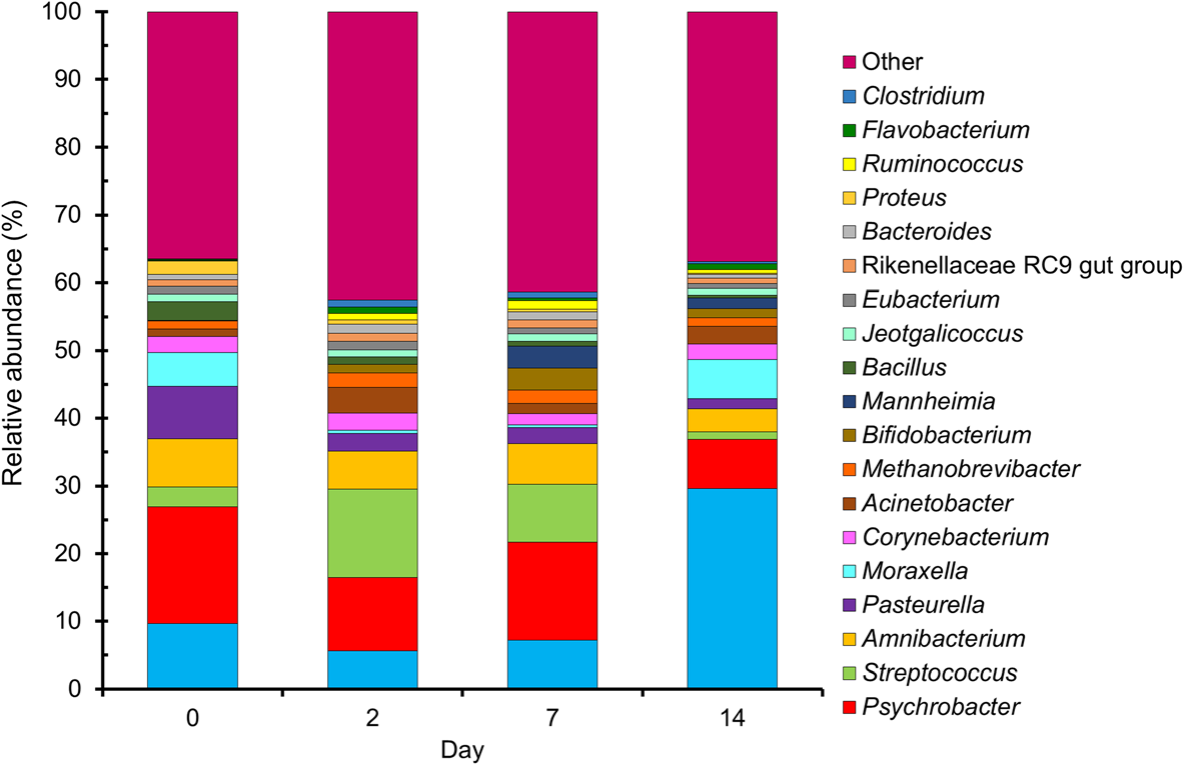
The 20 most relatively abundant archaeal and bacterial genera in the nasopharyngeal microbiota of cattle at days 0, 2, 7, and 14 of the study. For each sampling time *n* = 14 except day 0, where *n* = 13

Euryarchaeota (Additional file 5: Fig. S2). The 20 most relatively abundant genera are shown in Fig. 1. At the genus-level in particular, there was considerable inter-individual variability among cattle over the 14-d sampling period. For example, *Mycoplasma* and *Psychrobacter* each comprised greater than 12.0% of the total sequences overall, however, the range for *Mycoplasma* and *Psychrobacter* in individual cattle was between 0.003 to 86.0% and 0.55 to 90.0%, respectively. Intriguingly, a number of the most relatively abundant genera (*Methanobrevibacter*, *Bacteroides*, *Bifidobacterium*, *Clostridium*, RC9 gut group, and *Ruminococcus*) in the nasopharynx contain obligate anaerobic species. Despite the large number of OTUs present among all NP samples, 43.1% of the total 16S rRNA gene sequences were assigned to only 10 OTUs. There were 52 OTUs that were shared among all cattle at all sampling times (Additional file 6: Table S4), comprising what may be defined as the core microbiota in this context. As expected, this core microbiota consisted of many of the most relatively abundant OTUs such as those classified as *Mycoplasma*, *Psychrobacter*, *Amnibacterium*, and *Acinetobacter*.

### Temporal changes in the bovine nasopharyngeal microbiota

**Fig. 2:**
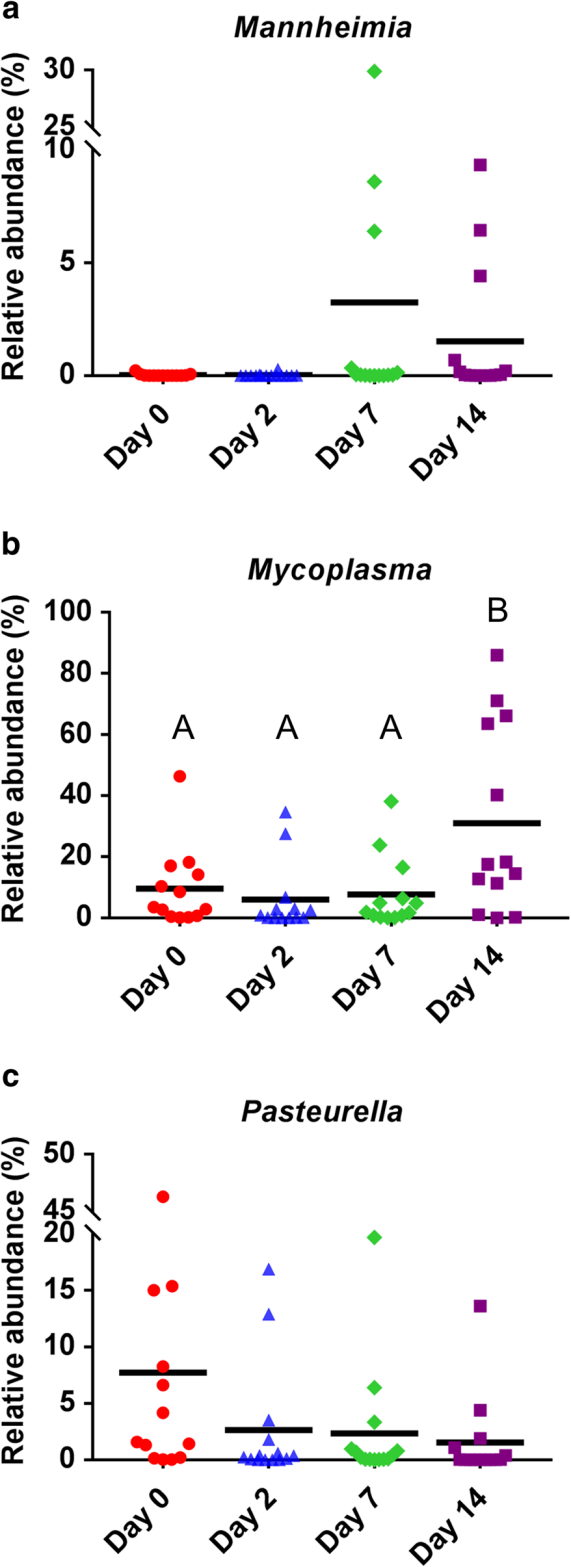
The relative abundance of bacterial genera associated with bovine respiratory disease in the nasopharyngeal microbiota of cattle. Cattle were sampled at a ranch (Day 0) prior to shipping to a feedlot and then 2, 7, and 14 days after feedlot placement. **a** *Mannheimia*, **b** *Mycoplasma*, **c** *Pasteurella*

**Fig. 3:**
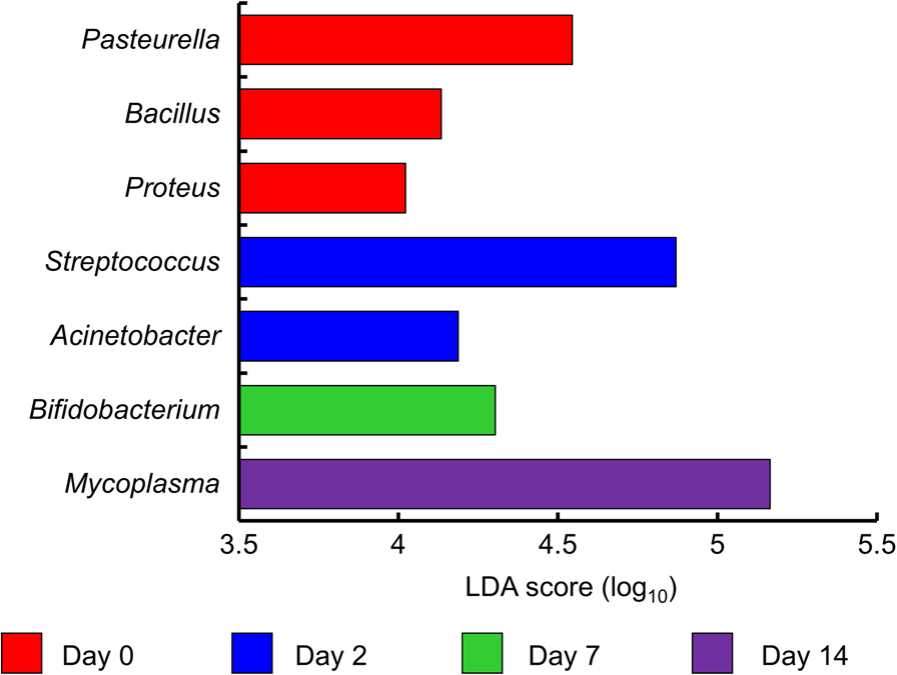
Genera enriched in the nasopharyngeal microbiota of cattle at specific sampling times, as determined using linear discriminant analysis effect size (LEfSe) analysis. Only genera with a linear discriminant analysis (LDA) score (log_10_) of > 4.0 are displayed. Cattle were sampled at a ranch (Day 0) prior to shipping to a feedlot and then 2, 7, and 14 days after feedlot placement. For each sampling time *n* = 14 except day 0, where *n* = 13

**Fig. 4:**
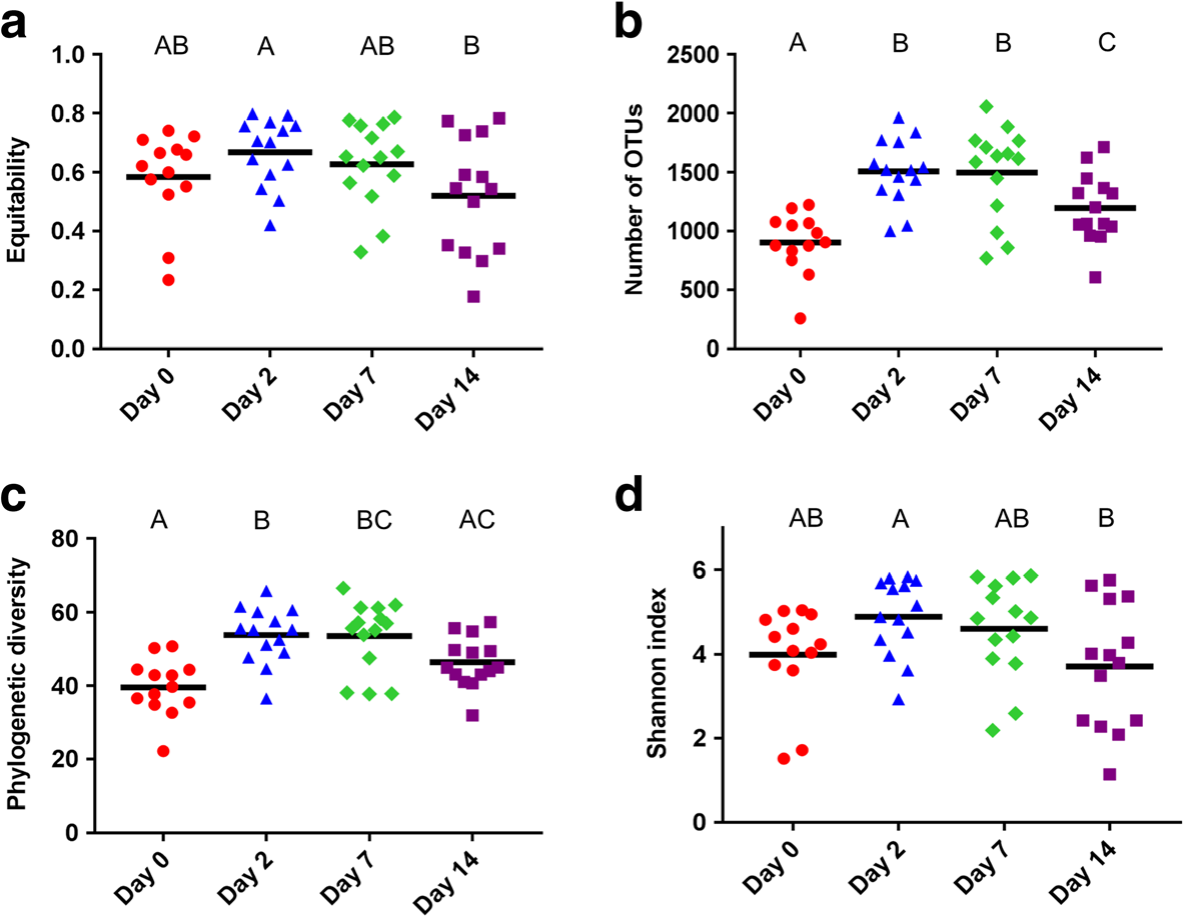
Alpha-diversity (within-sample) analysis of the nasopharyngeal microbiota of cattle over time. Cattle were sampled at a ranch (Day 0) prior to shipping to a feedlot and then 2, 7, and 14 days after feedlot placement. **a** equitability (evenness), **b** richness, **c** phylogenetic diversity (PD whole tree), and **d** the Shannon index. The solid, horizontal black line denotes the mean

A number of changes were noted in the NP microbiota of the cattle from the time they were sampled at the disease-free ranch (d 0) and then after transportation and holding at the feedlot over 14 d. With regard to BRD-associated bacteria, the relative abundance of *Mannheimia* did not change significantly over time (*P* > 0.05), although the NP microbiota of one heifer at d 7 was comprised of 30% *Mannheimia* (Fig. 2a). *Mycoplasma* was significantly more relatively abundant at d 14 compared to all other sampling times (*P* < 0.05; Fig. 2b). *Pasteurella* was higher at d 0, although at each of the other three sampling times at least one animal had a relative abundance of at least 10% *Pasteurella* in their nasopharynx and as a result of this variability this difference was not significant (Fig. 2c). However, when assessed using LEfSe *Pasteurella* was found to be enriched at d 0 compared to all other sampling times (Fig. 3). Six other bacterial genera were identified by LEfSe analysis as being strongly and significantly enriched at one specific sampling time (LDA score [log_10_] > 4.0; Fig. 3), including *Mycoplasma* at day 14. Among the other genera, *Bacillus*, *Streptococcus* and *Bifidobacterium* were notably more relatively abundant at days 0, 2, and 7, respectively. The number of OTUs (richness) and the phylogenetic diversity in each NP sample increased significantly from day 0 to days 2 and 7, however, by day 14, the phylogenetic diversity was similar to day 0 NP samples (Fig. 4). The evenness (equitability) and diversity (Shannon’s index) of the samples also varied over time, with greater evenness and Shannon diversity at d 2 compared to d 14.

**Fig. 5:**
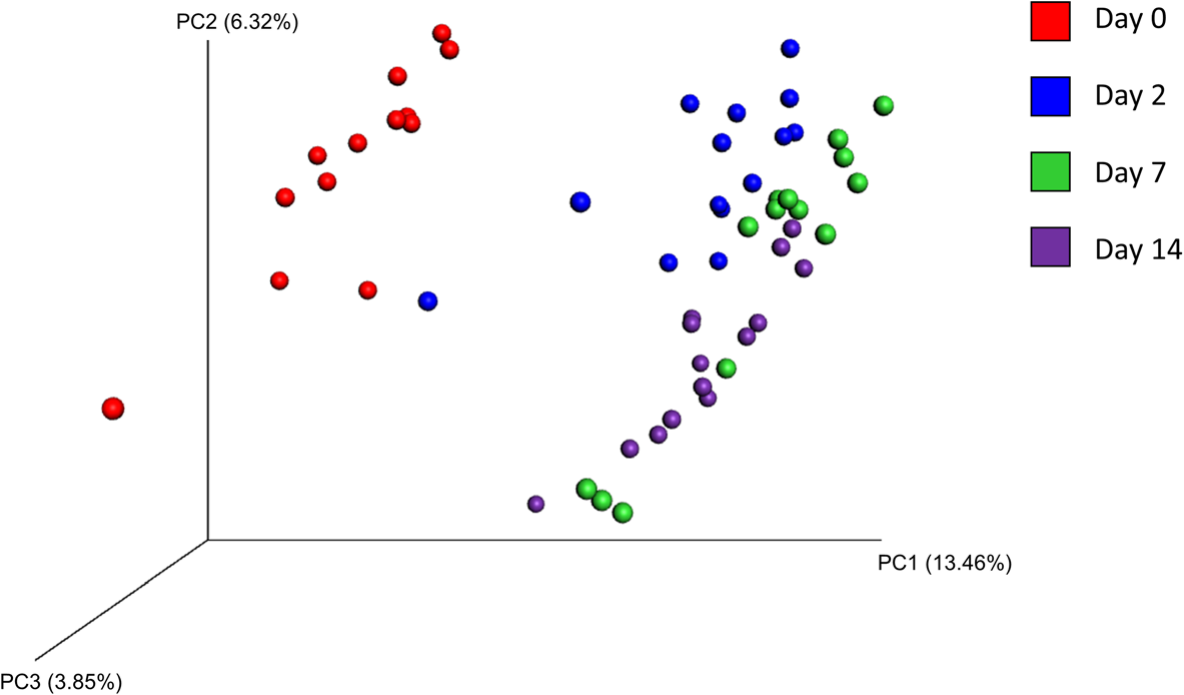
Principal coordinate analysis of the nasopharyngeal microbiota of cattle. Plots of the unweighted UniFrac distances by sampling time are shown. Cattle were sampled at a ranch (Day 0) prior to shipping to a feedlot and then 2, 7, and 14 days after feedlot placement. The percent variation explained by the principal coordinates is indicated on the axes

The NP microbiota structure also shifted significantly based on sampling time as assessed using unweighted UniFrac distances (*R*-value = 0.50; *P* < 0.001; Fig. 5). Samples taken prior to transport to the feedlot (day 0) were well separated from those at all other sampling times. When the NP community structure was determined using weighted UniFrac distances, there was still clustering by sampling time (*R*-value = 0.21; *P* < 0.001; Additional file 7: Fig. S3), however, the effect of time was not as strong as with the unweighted UniFrac distances. Thus, it would appear that the less relatively abundant taxa are most responsible for the shifts in the microbiota observed in Fig. 4.

The number of OTUs that were shared among all samples also differed at each time point. At day 0, prior to transport to the feedlot, there were 76 OTUs that were shared in the NP microbiota of all cattle. At day 2 there were 292 OTUs common to all NP samples, and 373 and 274 OTUs at days 7 and 14, respectively. As with the PCoA plot in Fig 5, this seems to indicate that not only was the NP microbiota shifting from day 0 to 2, but that it became more homogenous as well.

### Predicted functional potential for the bovine nasopharyngeal microbiota

Presently, the metagenome of the NP in cattle has not been characterized. Therefore, in the absence of metagenome sequencing, we used PICRUSt to predict the functional potential for the NP microbiota using 16S rRNA gene sequences. The NSTI (nearest sequenced taxon index) value was used to provide a measure of how well the OTUs in the NP samples were accounted for in the reference genome database. The NSTI for the NP samples was 0.07 ± 0.03 (SD), which is lower than the average NSTI for mammalian microbiomes (0.14), thus indicating that in relative terms, the OTUs in the NP samples in the present study are well represented in the reference genome database [26]. Overall, 74.4% of the reads matched a 16S rRNA gene sequence in the Greengenes database. Following normalization by 16S copy number, the OTUs were assigned to KEGG orthologies (KOs). To determine which OTUs might be excluded due to the use of the closed reference approach required for PICRUSt we also used the open reference OTU picking algorithm with the Greengenes 16S rRNA gene database (data not shown). Only four OTUs had an overall relative abundance of greater than 0.1% and these OTUs were classified only at the family level (*Chitinophagaceae*, *Microbacteriaceae*, *Moraxellaceae*, and *Weeksellaceae*).

**Fig. 6:**
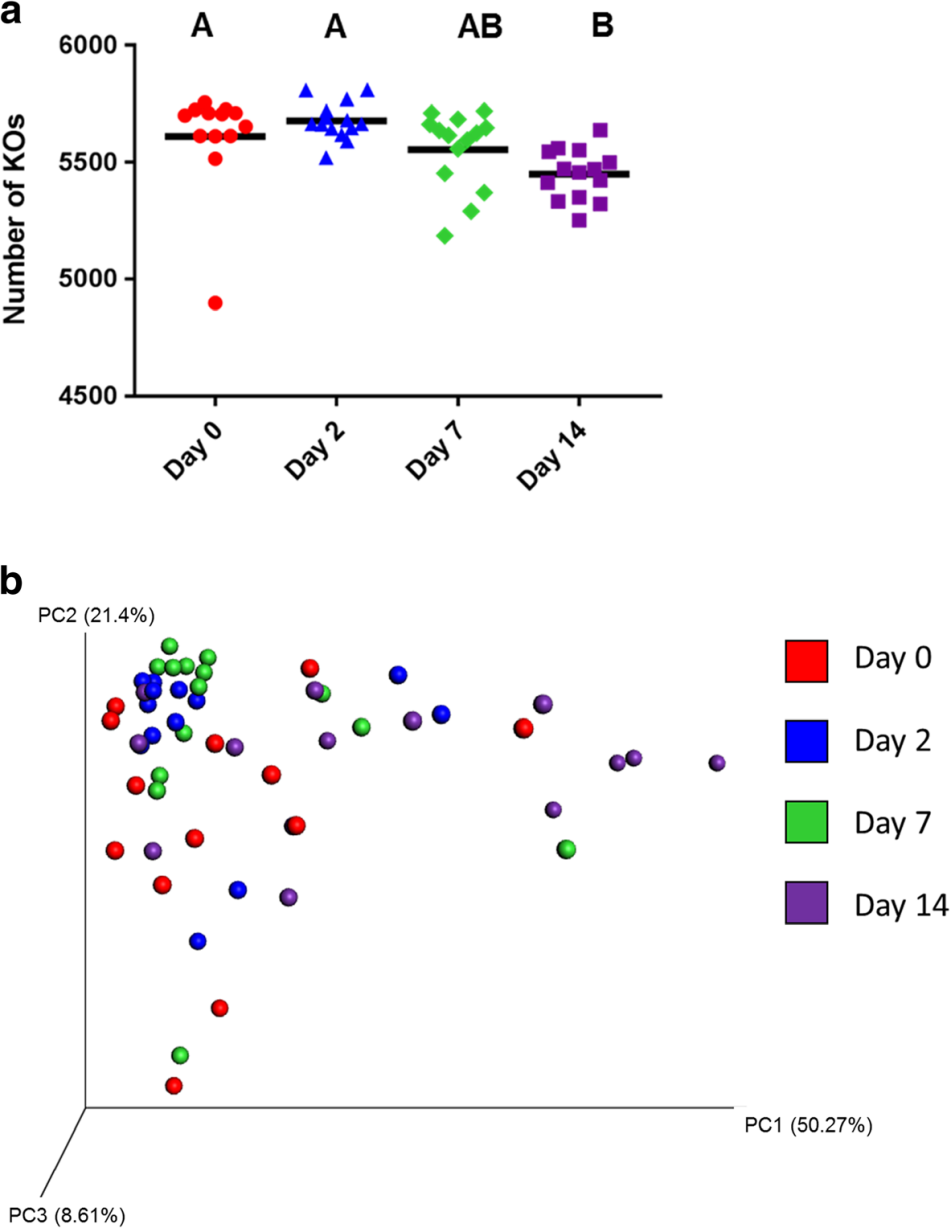
Functional predictions for the nasopharyngeal (NP) microbiota of cattle, as calculated using phylogenetic investigation of communities by reconstruction of unobserved states (PICRUSt). **a** Number of KEGG orthologies (KOs) per NP sample. **b** Principal coordinate analysis plot of Bray-Curtis distances for KOs in each NP sample by sampling time. Cattle were sampled at a ranch (Day 0) prior to shipping to a feedlot and then 2, 7, and 14 days after feedlot placement

When the number of KOs were compared by sampling time, there were significantly fewer KOs at day 14 than in day 0 and 2 samples (Fig. 6a; P < 0.05). These KOs were subsequently categorized into KEGG level 2 pathways, with membrane transport, carbohydrate metabolism, and amino acid metabolism as the three most relatively abundant pathways (Additional file 8: Fig. S4). There were 15 KEGG level 2 pathways that were differentially abundant among the four sampling times (Table 1; P < 0.05), the majority of which are involved in metabolism, with a number of them reduced at day 14 compared to day 0. In particular, there were fewer genes predicted to be involved in amino acid metabolism in the NP microbiome at day 14. Meanwhile, at day 14, genes required for translation, as well as replication and repair, were among those predicted to be more relatively abundant in the NP microbiome. Not surprisingly, given their relative abundance at day 14, *Mycoplasma* OTUs were the greatest contributors to the replication and repair pathway (data not shown). Bray-Curtis distances were used to compare changes in the predicted functional profile of the NP microbiome over time based on KOs (Fig. 6b). Samples did group together by sampling time (R = 0.16; *P* < 0.001), although not as strongly as with OTU-based clustering (Fig. 4).

**Table 1:** Differentially abundant KEGG level 2 pathways at each sampling time based on PICRUSt

KEGG level 2 pathway	Day 0	Day 2	Day 7	Day 14
Amino acid metabolism	10.03 ± 0.75A	9.99 ± 0.54A	9.80 ± 0.63AB	8.94 ± 1.22B
Biosynthesis of other secondary metabolites	0.74 ± 0.13AB	0.79 ± 0.09A	0.78 ± 0.11AB	0.66 ± 0.13B
Cell motility	2.00 ± 0.62A	1.92 ± 0.37AB	1.74 ± 0.39AB	1.41 ± 0.56B
Cellular processes and signaling	3.70 ± 0.54A	3.58 ± 0.37AB	3.62 ± 0.5A	3.01 ± 0.77B
Energy metabolism	5.95 ± 0.24AB	5.82 ± 0.2A	5.89 ± 0.2A	6.17 ± 0.34B
Genetic information processing	2.68 ± 0.13A	2.73 ± 0.11AB	2.79 ± 0.1AB	2.88 ± 0.2B
Metabolism	2.37 ± 0.12A	2.32 ± 0.13AB	2.29 ± 0.12AB	2.19 ± 0.21B
Metabolism of other amino acids	1.71 ± 0.12A	1.61 ± 0.07B	1.54 ± 0.09B	1.58 ± 0.09B
Metabolism of terpenoids and polyketides	1.82 ± 0.32A	1.8 ± 0.24A	1.7 ± 0.25AB	1.41 ± 0.47B
Nucleotide metabolism	4.15 ± 0.34A	4.31 ± 0.36AB	4.4 ± 0.33AB	4.67 ± 0.5B
Replication and repair	9.24 ± 1.05A	9.24 ± 0.9A	9.6 ± 1.04A	11.01 ± 1.85B
Signal transduction	1.61 ± 0.23A	1.54 ± 0.16A	1.44 ± 0.18AB	1.23 ± 0.36B
Transcription	2.46 ± 0.17A	2.71 ± 0.14B	2.7 ± 0.23B	2.44 ± 0.17A
Translation	6.61 ± 1.3A	6.61 ± 1.37A	6.96 ± 1.39A	8.88 ± 2.54B
Xenobiotics biodegradation and metabolism	2.61 ± 0.32A	2.39 ± 0.26AB	2.12 ± 0.24B	2.07 ± 0.48B

Mean relative abundance ± standard deviation. Different uppercase letters in rows are significantly different (*P* < 0.05)

## Discussion

Understanding how the bovine NP microbiota changes following introduction to a feedlot is important due to the fact that beef cattle are most susceptible to BRD during this period of time. The transportation [8] of cattle to the feedlot has been associated with an increased risk of developing BRD, and may be related to physiological changes (i.e. stress) that allow for pathogen proliferation and infection [33]. In addition to changes in pathogen prevalence, it is possible that alterations in other community bacteria affect BRD pathogen growth. Aerobic bacteria from the bovine respiratory tract have previously been shown to both enhance and inhibit the growth of *M. haemolytica* and *P. multocida* in vitro [34], thus the total NP microbiota may be critical to disease susceptibility. In the present study, it was determined that the NP microbiota of calves undergoes numerous alterations, both in community membership and structure, following the first 14 days in the feedlot environment. An advantage of our study was using cattle with a known history and from a well-established disease-free herd that were managed under conditions limiting disruptions of the NP microbiota.

### The bovine nasopharyngeal microbiota

In agreement with previous work on the bovine NP microbiota, the bacterial phyla Proteobacteria, Firmicutes, Tenericutes, and Actinobacteria were relatively abundant at all sampling times [1–3, 35]. One of the more surprising findings was the detection of Euryarchaeota 16S rRNA gene sequences at an overall relative abundance of 1.8%. Although it is not known if these sequences represented viable organisms, the Euryarchaeota phylum contained the methanogenic Archaea *Methanobrevibacter*, which are most often associated with the rumen and gastrointestinal tract where they can comprise 2 to 6% of the total microorganisms [36, 37]. Given that methanogens are considered to be strict anaerobes, it may be that rather than colonizing the nasopharynx, these microbes are continuously being re-introduced through rumination and eructation.

Similar to the *Methanobrevibacter*, the obligate anaerobic genera *Bacteroides*, *Bifobacterium*, *Clostridium*, and *Ruminococcus* were all relatively abundant in the NP samples. Recently, *Bacteroides* and *Ruminococcus*, along with other obligate anaerobic genera such as *Fusobacterium*, *Porphyromonas*, and *Prevotella*, were reported to be present in the nasopharynx of dairy cattle [35]. *Fusobacterium* has also been isolated from the bovine respiratory tract [38]. Although the nasopharynx is typically described as being predominantly aerobic these results suggest that anaerobic bacteria may form a significant portion of the NP microbiota in cattle.

Although there was considerable inter-individual variability, many of the relatively abundant genera found in the NP microbiota of the current study were also identified previously as being among the most relatively abundant in the NP of feedlot [1, 3] and dairy cattle [35]. In addition, of the 20 most relatively abundant genera, *Acinetobacter*, *Bacillus*, *Corynebacterium*, *Mannheimia*, *Moraxella*, *Pasteurella*, *Proteus*, *Psychrobacter*, and *Streptococcus* have all been isolated from the bovine nasopharynx using culture-based methods [1, 5, 39]. *Mycoplasma*, which is typically more difficult to isolate, is also frequently identified when appropriate culture-conditions are employed [40, 41]. Interestingly, *Amnibacterium* was the fourth most relatively abundant genus overall, although this was largely a result of 10 samples that had a relative abundance of greater than 10%. This genus, which belongs to the *Microbacteriaceae* family, has only been recently described [42] and included in the SILVA database, explaining its previous absence from characterizations of the NP microbiota. Species of *Amnibacterium* have been identified in water [42] and soil [43] perhaps indicating that the environment was the source of this genus in nasopharyngeal samples.

### The effect of feedlot placement on the bovine nasopharyngeal microbiota

The structure of the NP microbiota continued to shift during the 14-d monitoring period, however, the changes observed from day 0 to day 2, as determined using unweighted UniFrac distances (Fig. 5), were particularly noticeable. The microbial richness and phylogenetic diversity also both significantly increased following transport to the feedlot (day 2). These early alterations in the NP microbiota may be expected given the number of changes in the external environment of the cattle during this period. For example, cattle are potentially exposed to new microbes in the feedlot pens, including those in water and feed which have different sources compared to the cow/calf farm. In addition, feedlot transport is a known stressor for cattle, influencing immune function through higher cortisol levels, altered acute-phase protein response, and modifying the concentrations of other white blood cells, which may in turn alter the microbiota [44–46]. Overall, the changes in the microbial community structure were mostly driven by the less relatively abundant taxa given the differences observed between the unweighted and weighted UniFrac distances. The NP microbiota did appear to become more homogenous over the 14-d study period as the number of OTUs shared by all animals at each sample time increased from only 76 at day 0 to an average of 313 at the days 2, 7, and 14.

In terms of membership, most striking was the enrichment of *Mycoplasma* in day 14 samples (Figs. 2 and 3). At this time point, four animals actually had a NP microbiota that was comprised of greater than 63% *Mycoplasma*. We have previously observed an increase in *Mycoplasma* 60 d after feedlot entry [1] indicating that the feedlot environment, and its associated stressors, may provide conditions that allow for the proliferation of *Mycoplasma* in the nasopharynx. Based on the present study, it would appear that changes in the NP microbiota that allow *Mycoplasma* to proliferate occur within the first 14 d of feedlot placement. Further classification of the most relatively abundant OTUs identified as *Mycoplasma* revealed that they were evenly split between the species *M. bovirhinis* and *M. dispar*. Although *M. bovirhinis* and *M. dispar* are isolated from diseased as well as healthy cattle, *M. bovis* is the primary *Mycoplasma* sp. associated with BRD [47, 48]. Because of its importance in chronic pneumonia, it would be of interest to determine whether *M. bovis* is similarly capable of rapid proliferation and occupying a larger relative proportion of the NP microbiota, should it be present to colonize the nasopharynx at feedlot entry. In a previous study, the prevalence of *M. bovis* in bronchoalveolar lavage fluid increased from 1.7% at feedlot entry to 72.2% 15 days after mixing and placement of cattle in a commercial feedlot [49]. Although the authors did not report on relative abundance, it is apparent that *M. bovis* is capable of spreading amongst cattle when a source of this opportunistic pathogen is available.

*Pasteurella* spp., which were enriched in the nasopharynx of cattle prior to transport to the feedlot, were identified by BLASTn to be *P. multocida*, a bacterial species commonly associated with BRD. Despite the presence of these species, and also *M. haemolytica* at a lower relative abundance, the cattle all remained healthy throughout the study, thereby confirming that *Mycoplasma* spp., *P. multocida*, and *M. haemolytica* are natural inhabitants of the NP tract. This observation is further supported by the presence of these bacteria in the cattle prior to shipping to the feedlot, as we used cattle from a closed and disease-free herd that has been maintained in this state for over 30 years. It is of particular interest that transportation to a feedlot did not result in proliferation of *P. multocida* and *M. haemolytica*, the latter being the opportunistic pathogen most often associated with BRD cases. In contrast to this, increased shedding of *M. haemolytica* has previously been described as a result from stress associated with cattle transportation, although in that study, the distance travelled was 1,600 km and the authors used recently weaned calves [50] which may have increased predisposition to pathogen growth.

### The functional metagenome of the bovine nasopharnx

We used PICRUSt to predict the functional composition of the NP microbiome based on 16S rRNA gene sequences. The gene content of the NP microbial community was then inferred and categorized into KOs, which are further classified into KEGG level 2 pathways. Overall, level 2 KEGG pathways associated with membrane transport, carbohydrate metabolism, and amino acid metabolism were the most relatively abundant in the NP microbiome of all cattle. Given that amino acids, carbohydrates, lipids, nucleic acids, and proteins are all found within the mucosa of the respiratory tract, it is reasonable to expect that pathways responsible for their metabolism would also be the most abundant in the microbiome [51].

There were fifteen level 2 KEGG pathways that were differentially abundant among the four sampling times, with most of these differences occurring between day 0 and 14 NP samples (Table 1). Most notably, amino acid metabolism, cellular processes and signaling, and cell motility pathways were predicted to be enriched among the day 0 samples compared with day 14. KEGG pathways associated with replication and repair, as well as translation, were among those that were predicted to be more relatively abundant at day 14. *Mycoplasma* spp. which were more relatively abundant at day 14, were also the greatest contributors to these two pathways at day 14. The number of KOs among sampling times did not differ significantly, although there was clustering by sampling time based on Bray-Curtis distances.

## Conclusions

We used a disease-free and closed herd in the present study which allowed us to investigate the dynamics of the NP microbiota of beef cattle following transport and arrival at a feedlot without potential confounding factors such as antimicrobial use and vaccination. Overall, it is evident that the bovine NP microbiota undergoes a number of significant and relatively rapid changes in terms of structure and membership following arrival at the feedlot. Introduction to the feedlot environment increased both the richness and phylogenetic diversity of the NP microbiota within the first 48 h of arrival. Functional analysis using PICRUSt also predicted that pathways associated with metabolism and DNA replication and repair were differentially abundant between days 0 and 14. Although feedlot placement did not increase BRD-associated bacterial pathogens, with the exception of *Mycoplasma*, the relative instability of the NP microbiota immediately following feedlot placement may help explain why cattle are most susceptible to BRD during this period. Other factors such as age, vaccination status, antimicrobial use history, and mingling also need to be investigated to better define parameters throughout the beef continuum that can alter the NP microbiota, and in particular, opportunistic pathogens.

## Additional files

Additional file 1: Figure S1. A sagittal midline view of a bovine head (adult animal ≥ 36 months old). The image indicates that swabs used in the current study (27 cm length) were able to reach the nasopharynx of 8-month old calves during sampling. (TIF 8079 kb)

Additional file 2: Table S1. Multiplex primers used for the identification of bovine respiratory disease-associated bacteria. (DOCX 17 kb)

Additional file 3: Table S2. Calves positive for *Pasteurella multocida* by culturing of nasopharyngeal swabs. (DOCX 12 kb)

Additional file 4: Table S3. BLASTn results for OTUs classified by the UCLUST consensus taxonomy assigner as *Pasteurella, Mannheimia, or Mycoplasma*. (DOCX 17 kb)

Additional file 5 Figure S2. The six most relatively abundant archaeal and bacterial phyla in the nasopharyngeal microbiota of cattle at days 0, 2, 7, and 14 of the study. For each sampling time *n* = 14 except day 0, where *n* = 13. (TIF 1601 kb)

Additional file 6: Table S4. OTUs found in all nasopharyngeal samples at all sampling times. (XLSX 15 kb)

Additional file 7: Figure S3. Principal coordinate analysis plots of the weighted UniFrac distances by sampling time. Day 0 samples were taken prior to transport to the feedlot. The percent variation explained by the principal coordinates is indicated on the axes. (TIF 671 kb)

Additional file 8: Figure S4. Phylogenetic investigation of communities by reconstruction of unobserved states (PICRUSt) analysis showing the 10 most relatively abundant level 2 KEGG pathways in the predicted metagenome at each sampling time. (TIF 1038 kb)

## Abbreviations

ANOSIM: Analysis of similarities
ANOVA: Analysis of variance
BHI: Brain heart infusion
BRD: Bovine respiratory disease
FDR: False discovery rate
DNA: Deoxyribonucleic acid
EDTA: Ethylenediaminetetraacetic acid
HSD: Honestly significant difference
KEGG: Kyoto encyclopedia of genes
KO: KEGG orthologies
LDA: Linear discriminant analysis
LEfSe: Linear discriminant analysis effect size
NP: Nasopharyngeal
NSTI: Nearest sequenced taxon index
OTU: Operational taxonomic units
PCoA: Principal coordinate analysis
PCR: Polymerase chain reaction
PD: Phylogenetic diversity
PICRUSt: Phylogenetic investigation of communities by reconstruction of unobserved states
TSA: Tryptic soy agar
QIIME: Quantitative insights into microbial ecology
