# Erratum to: The nasopharyngeal microbiota of beef cattle before and after transport to a feedlot

**DOI:** 10.1186/s12866-017-1007-5

**Published:** 2017-04-20

**Authors:** Devin B. Holman, Edouard Timsit, Samat Amat, D. Wade Abbott, Andre G. Buret, Trevor W. Alexander

**Affiliations:** 1 United States Department of Agriculture, National Animal Disease Center, Agricultural Research Service, Ames, USA; 2 Department of Production Animal Health, University of Calgary, Calgary, Canada; 3 Lethbridge Research Centre, Agriculture and Agri-Food Canada, Lethbridge, Canada; 4 Department of Biological Sciences, University of Calgary, Calgary, Canada

## Erratum

Upon publication of the original article [1] it was noted that our Standard Copyright was incorrectly attributed to this manuscript. The correct copyright attributed to this paper is as follows:

“© Her Majesty the Queen in Right of Canada 2017. This article is distributed under the terms of the Creative Commons 4.0 Attribution License (http://creativecommons.org/licenses/by/4.0/), which permits unrestricted use, distribution, and reproduction in any medium, provided you give appropriate credit to the original author(s) and the source, provide a link to the Creative Commons license, and indicate if changes were made. The Creative Commons Public Domain Dedication waiver (http://creativecommons.org/publicdomain/zero/1.0/) applies to the data made available in this article, unless otherwise stated.”

This has now also been corrected in the original article.

## References

[1] Holman DB, Timsit E, Amat S, Abbott DW, Buret AG, Alexander TW (2017). The nasopharyngeal microbiota of beef cattle before and after transport to a feedlot. BMC Microbiol.

