# Supplementary figures and images for: The nasopharyngeal microbiota of beef cattle before and after transport to a feedlot
Source: BMC Microbiol. 2017 Mar 22;17:70. doi: 10.1186/s12866-017-0978-6 (PMC5361731; doi:10.1186/s12866-017-0978-6)

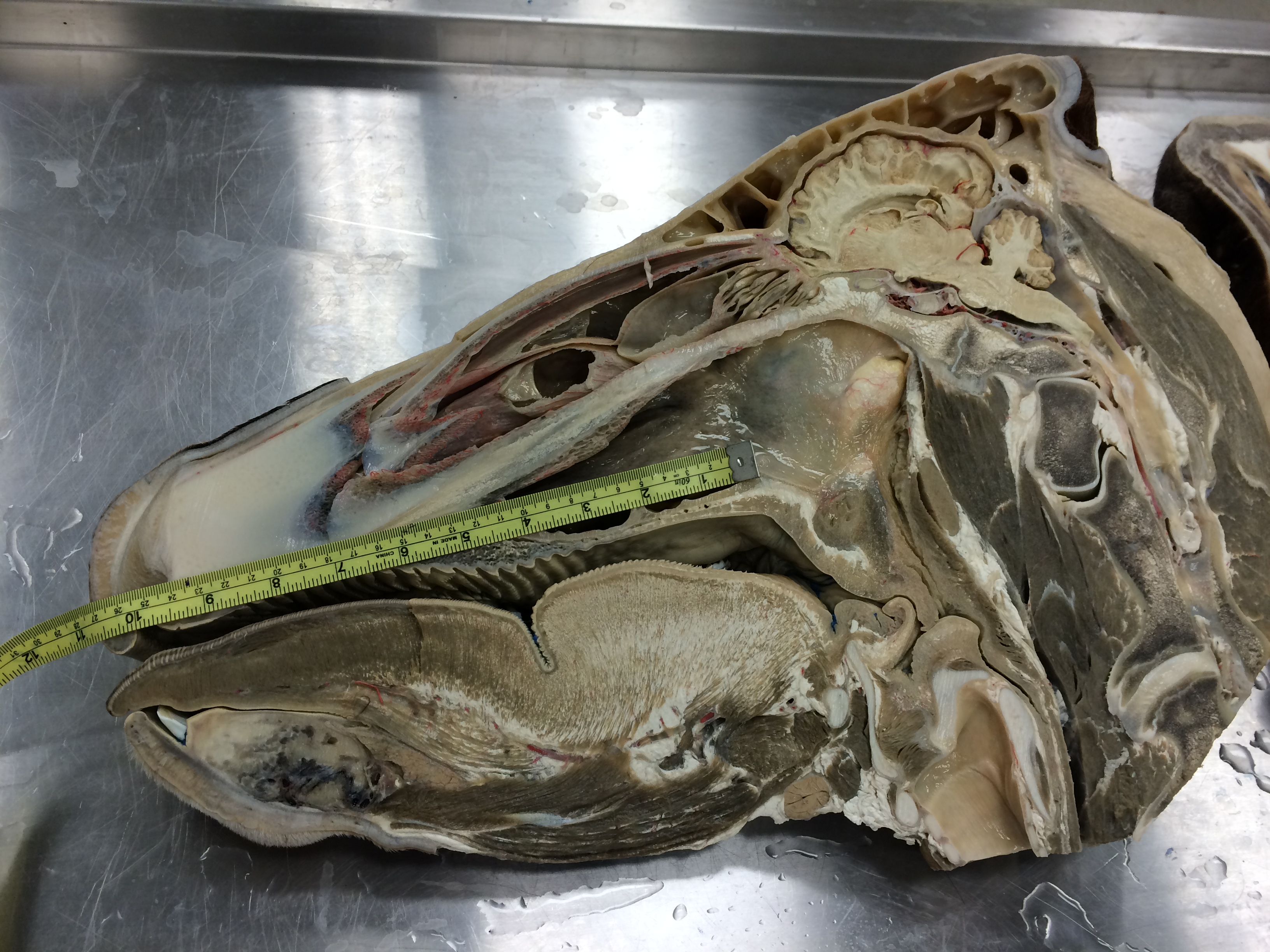

Supplement: Supplementary file 1 — A sagittal midline view of a bovine head (adult animal ≥ 36 months old). The image indicates that swabs used in the current study (27 cm length) were able to reach the nasopharynx of 8-month old calves during sampling. (TIF 8079 kb) [file 12866_2017_978_MOESM1_ESM.tif]

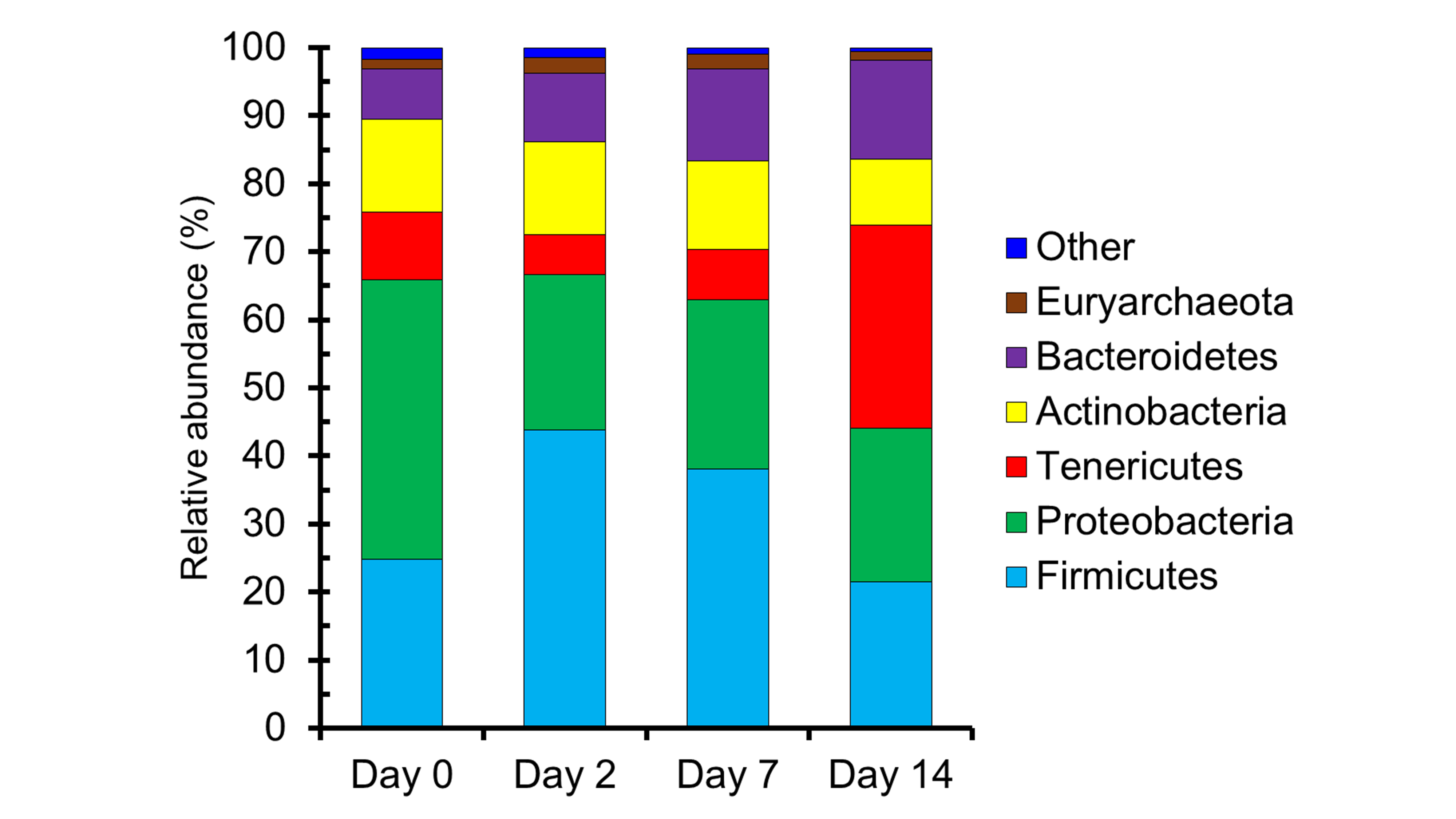

Supplement: Supplementary file 5 — The six most relatively abundant archaeal and bacterial phyla in the nasopharyngeal microbiota of cattle at days 0, 2, 7, and 14 of the study. For each sampling time n = 14 except day 0, where n = 13. (TIF 1601 kb) [file 12866_2017_978_MOESM5_ESM.tif]

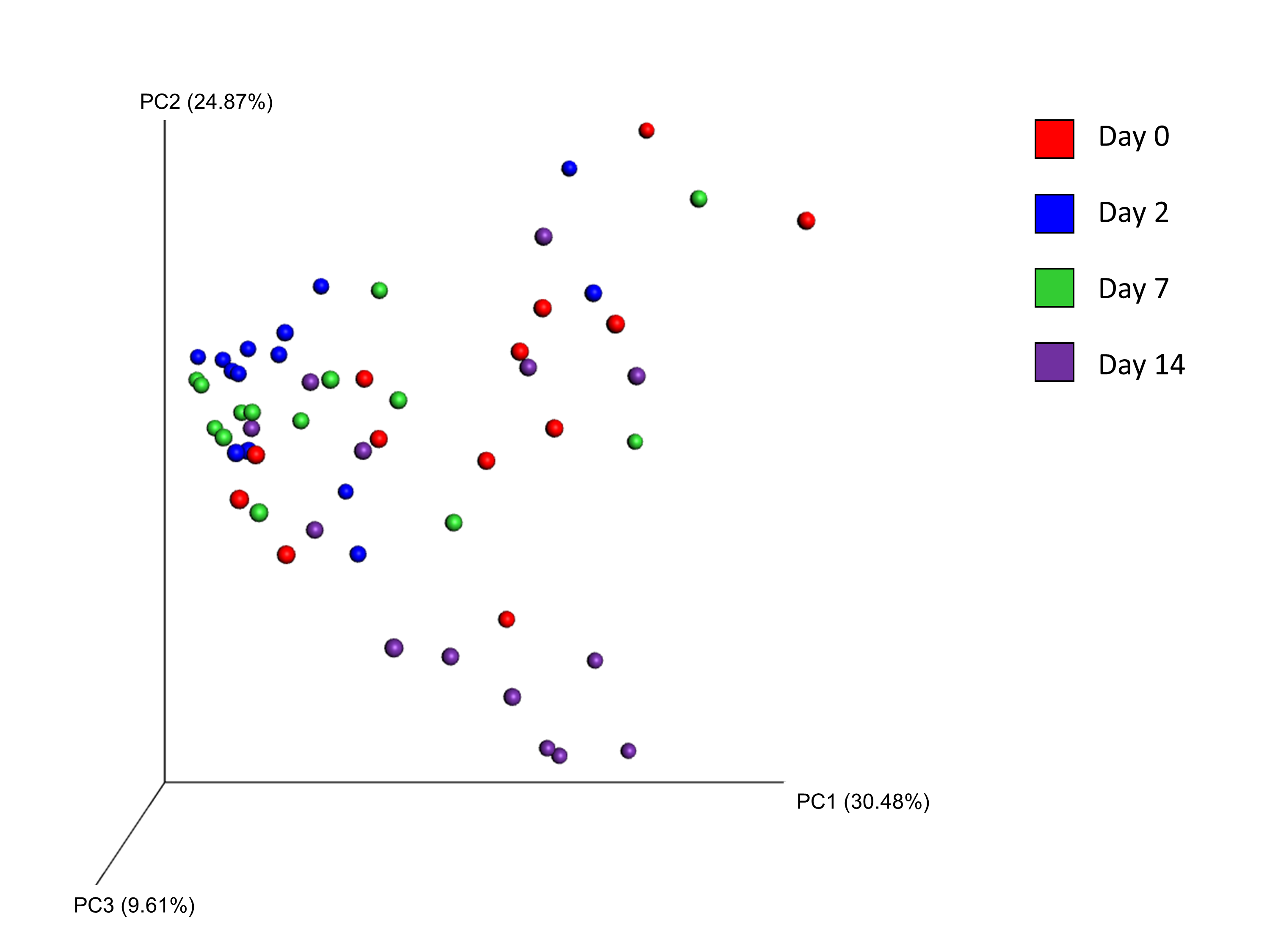

Supplement: Supplementary file 7 — Principal coordinate analysis plots of the weighted UniFrac distances by sampling time. Day 0 samples were taken prior to transport to the feedlot. The percent variation explained by the principal coordinates is indicated on the axes. (TIF 671 kb) [file 12866_2017_978_MOESM7_ESM.tif]

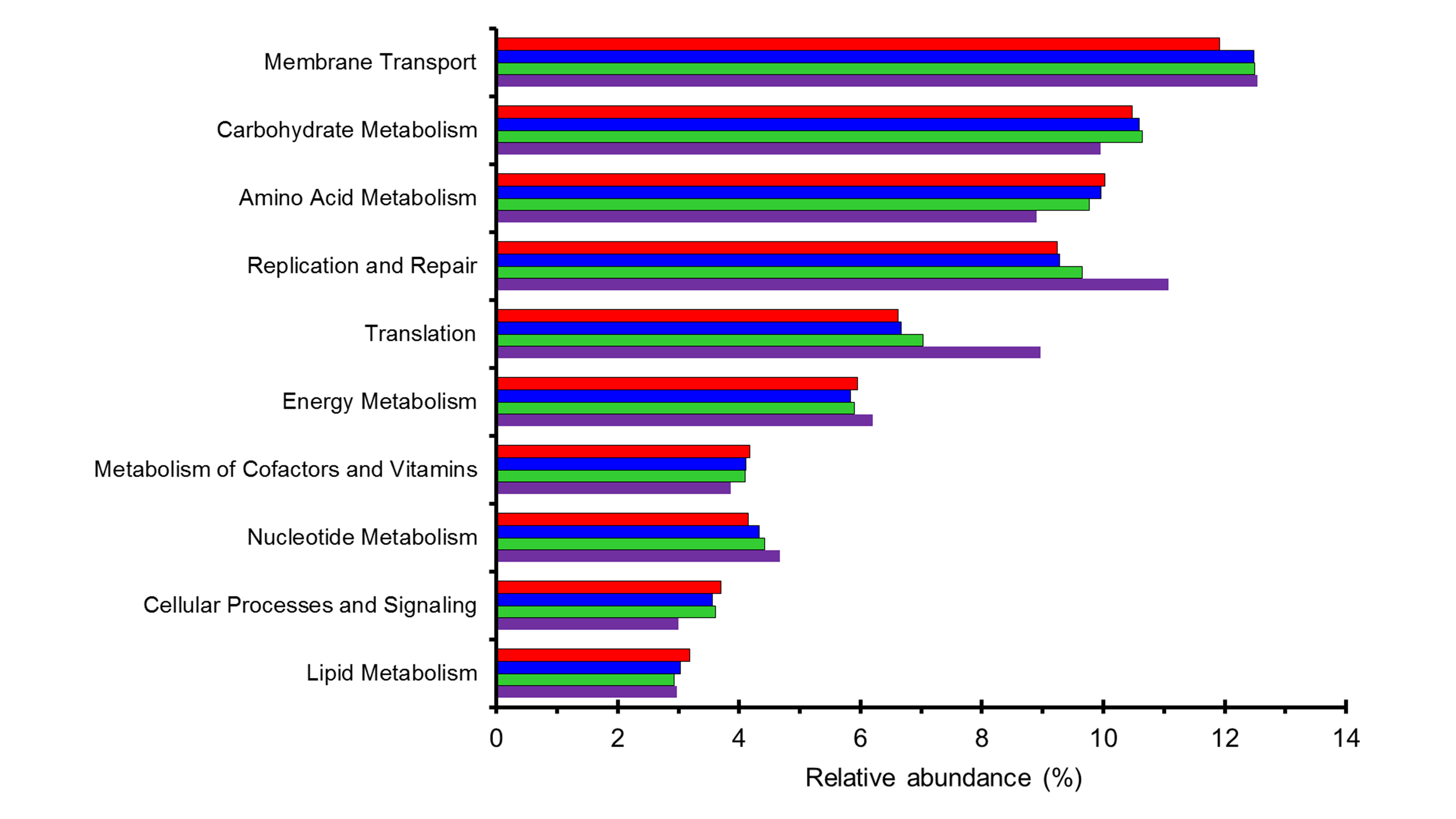

Supplement: Supplementary file 8 — Phylogenetic investigation of communities by reconstruction of unobserved states (PICRUSt) analysis showing the 10 most relatively abundant level 2 KEGG pathways in the predicted metagenome at each sampling time. (TIF 1038 kb) [file 12866_2017_978_MOESM8_ESM.tif]
