# Supplementary material for: The nasopharyngeal microbiota of beef cattle before and after transport to a feedlot
Source: BMC Microbiol. 2017 Mar 22;17:70. doi: 10.1186/s12866-017-0978-6 (PMC5361731; doi:10.1186/s12866-017-0978-6)
Supplement: Supplementary file 2 — Multiplex primers used for the identification of bovine respiratory disease-associated bacteria. (DOCX 17 kb) [file 12866_2017_978_MOESM2_ESM.docx]

| **Table S1.** Multiplex primers used for the identification of bovine respiratory disease-associated bacterial isolates. | | |  |
| --- | --- | --- | --- |
| **Target** | **Sequence (5′ to 3′)** | **Amplicon size (bp)** | **Annealing temp (°C)** |
| *H. somni* | F-GAAGGCGATTAGTTTAAGAG | 400 | 55[1] |
|  | R-TTCGGGCACCAAGTRTTCA |  |  |
| *M. haemolytica* | F-GTCCCTGTGTTTTCATTATAAG | 385 | 58[2] |
|  | R-CACTCGATAATTATTCTAAATTAG |  |  |
| *P. multocida* | F-CGAGCAAGCACAATTACATTATGG | 90 | 52[3] |
|  | R-CACCGTCAAATTCCTGTGGATAAC |  |  |

1. Angen O, Ahrens P, Tegtmeier C: Development of a PCR test for identification of *Haemophilus somnus* in pure and mixed cultures. Vet Microbiol. 1998; 63:39-48.

2. Alexander TW, Cook SR, Yanke LJ, Booker CW, Morley PS, Read RR, Gow SP, McAllister TA: A multiplex polymerase chain reaction assay for the identification of *Mannheimia haemolytica*, *Mannheimia glucosida* and *Mannheimia ruminalis*. Vet Microbiol. 2008; 130:165-175.

3. Angen O, Thomsen J, Larsen LE, Larsen J, Kokotovic B, Heegaard PM, Enemark JM: Respiratory disease in calves: microbiological investigations on trans-tracheally aspirated bronchoalveolar fluid and acute phase protein response. Vet Microbiol. 2009; 137:165-171.
