# Supplementary material for: The nasopharyngeal microbiota of beef cattle before and after transport to a feedlot
Source: BMC Microbiol. 2017 Mar 22;17:70. doi: 10.1186/s12866-017-0978-6 (PMC5361731; doi:10.1186/s12866-017-0978-6)
Supplement: Supplementary file 3 — Calves positive for Pasteurella multocida by culturing of nasopharyngeal swabs. (DOCX 12 kb) [file 12866_2017_978_MOESM3_ESM.docx]

**Table S2**. Heifers positive for *Pasteurella multocida* by culturing of nasopharyngeal swabs^1^.

| Sampling time (day) | Animals positive |
| --- | --- |
| 0 | 3, 9, 17, 40, 43 |
| 2 | 3, 9, 35, 38, 42 |
| 7 | 3,17, 35, 38, 43 |
| 14 | 43 |

^1^ Numbers represent individual animal number. *Histophilus* *somni* and *Mannheimia haemolytica* were not isolated from any animals during the study.
