# Supplementary material for: The nasopharyngeal microbiota of beef cattle before and after transport to a feedlot
Source: BMC Microbiol. 2017 Mar 22;17:70. doi: 10.1186/s12866-017-0978-6 (PMC5361731; doi:10.1186/s12866-017-0978-6)
Supplement: Supplementary file 4 — BLASTn results for OTUs classified by the UCLUST consensus taxonomy assigner as Pasteurella, Mannheimia, or Mycoplasma. (DOCX 17 kb) [file 12866_2017_978_MOESM4_ESM.docx]

**Table S3**. BLASTn results for OTUs classified by the UCLUST consensus taxonomy assigner as *Pasteurella, Mannheimia*, or *Mycoplasma*. For *Mycoplasma* only the five most relatively abundant OTUs are included as they represented 99.1% of all *Mycoplasma* sequences.

| **OTU ID** | **OTU relative abundance (%)** | **Silva taxonomy (genus)** | **Top BLASTn hit (species)** | **E -value** | **Identity** | **Accession** |
| --- | --- | --- | --- | --- | --- | --- |
| EF579813.1.1364 | 1.54192 | *Pasteurella* | *Pasteurella multocida* subsp. *multocida* HB01, complete genome | 8.00E-129 | 100% | CP006976.1 |
| KF066487.1.1361 | 0.83604 | *Pasteurella* | *Pasteurella multocida* subsp. *multocida* HB01 | 3.00E-128 | 100% | CP006976.1 |
| GU177867.1.1309 | 0.73560 | *Pasteurella* | *Pasteurella multocida* subsp. *multocida* HB01 | 6.00E-130 | 100% | CP006976.1 |
| JF186299.1.1361 | 0.03020 | *Pasteurella* | *Pasteurella multocida* subsp. *multocida* HB01 | 1.00E-126 | 99% | CP006976.1 |
| CDON01000005.1.1449 | 0.00949 | *Pasteurella* | *Pasteurella multocida* subsp. *multocida* HB01 | 1.00E-127 | 99% | CP006976.1 |
| DQ851143.1.1540 | 0.00734 | *Pasteurella* | *Pasteurella multocida* subsp. *multocida* HB01 | 4.00E-127 | 99% | CP006976.1 |
| DQ666543.1.1362 | 0.00478 | *Pasteurella* | *Pasteurella multocida* subsp. *multocida* HB01 | 3.00E-128 | 99% | CP006976.1 |
| New.ReferenceOTU85 | 0.00450 | *Pasteurella* | *Pasteurella multocida* subsp. *multocida* HB01 | 2.00E-110 | 96% | CP006976.1 |
| AY362914.1.1363 | 0.00406 | *Pasteurella* | *Pasteurella multocida* subsp. *multocida* HB01 | 2.00E-129 | 99% | CP006976.1 |
| HM746988.1.1391 | 0.00364 | *Pasteurella* | *Pasteurella multocida* subsp. *multocida* HB01 | 8.00E-129 | 99% | CP006976.1 |
| JQ993874.1.1421 | 0.00276 | *Pasteurella* | *Pasteurella multocida* subsp. *multocida* HB01 | 2.00E-129 | 99% | CP006976.1 |
| KM079615.1.1441 | 0.00257 | *Pasteurella* | *Pasteurella multocida* subsp. *multocida* HB01 | 1.00E-127 | 99% | CP006976.1 |
| AF053902.1.1504 | 0.00254 | *Pasteurella* | *Pasteurella multocida* subsp. *multocida* HB01 | 3.00E-128 | 99% | CP006976.1 |
| ARFN01000005.1.1311 | 0.00240 | *Pasteurella* | *Pasteurella multocida* subsp. *multocida* HB01 | 8.00E-129 | 99% | CP006976.1 |
| New. ReferenceOTU213 | 0.00204 | *Pasteurella* | *Pasteurella multocida* subsp. *multocida* HB01 | 1.00E-112 | 96% | CP006976.1 |
| FJ463886.1.1365 | 0.00163 | *Pasteurella* | *Pasteurella multocida* subsp. *multocida* HB01 | 8.00E-129 | 99% | CP006976.1 |
| New.ReferenceOTU357 | 0.00144 | *Pasteurella* | Uncultured bacterium clone ncd294g08c1 16S ribosomal RNA gene, partial sequence | 6.00E-100 | 93% | HM272342.1 |
| AY362887.1.1362 | 0.00141 | *Pasteurella* | *Pasteurella multocida* subsp. *multocida* HB01 | 3.00E-128 | 99% | CP006976.1 |
| HK240617.1.1279 | 0.00075 | *Pasteurella* | *Pasteurella multocida* subsp. *multocida* HB01 | 8.00E-129 | 99% | CP006976.1 |
| New.ReferenceOTU3183 | 0.00058 | *Pasteurella* | *Pasteurella multocida* subsp. *multocida* HB01 | 4.00E-112 | 96% | CP006976.1 |
| JQ456522.1.1391 | 0.00055 | *Pasteurella* | *Pasteurella multocida* subsp. *multocida* HB01 | 8.00E-129 | 99% | CP006976.1 |
| New.ReferenceOTU724 | 0.00055 | *Pasteurella* | *Pasteurella multocida* subsp. *multocida* HB01 | 4.00E-112 | 96% | CP006976.1 |
| JF221861.1.1361 | 0.00047 | *Pasteurella* | *Pasteurella multocida* subsp. *multocida* HB01 | 6.00E-125 | 99% | CP006976.1 |
| New.ReferenceOTU4626 | 0.00047 | *Pasteurella* | *Pasteurella multocida* subsp. *multocida* HB01 | 2.00E-100 | 93% | CP006976.1 |
| New.ReferenceOTU4614 | 0.00044 | *Pasteurella* | *Pasteurella multocida* subsp. *multocida* HB01 | 6.00E-115 | 96% | CP006976.1 |
| AY172726.1.1451 | 0.00041 | *Pasteurella* | *Pasteurella multocida* subsp. *multocida* HB01 | 1.00E-127 | 99% | CP006976.1 |
| AY508821.1.1365 | 0.00041 | *Pasteurella* | *Pasteurella multocida* subsp. *multocida* HB01 | 4.00E-127 | 99% | CP006976.1 |
| HK240614.1.1296 | 0.00036 | *Pasteurella* | *Pasteurella multocida* subsp. *multocida* HB01 | 4.00E-127 | 99% | CP006976.1 |
| New.ReferenceOTU4753 | 0.00028 | *Pasteurella* | *Pasteurella multocida* subsp. *multocida* HB01 | 1.00E-96 | 92% | CP006976.1 |
| AY465370.1.1362 | 1.33963 | *Mannheimia* | *Mannheimia varigena* strain HSR GLF138 16S ribosomal RNA gene, partial sequence | 4.00E-127 | 99% | KM389532.1 |
| AY362917.1.1362 | 0.05755 | *Mannheimia* | *Mannheimia haemolytica* strain MB1401 16S ribosomal RNA gene, partial sequence | 6.00E-130 | 100% | KT013278.1 |
| DQ666553.1.1362 | 0.03326 | *Mannheimia* | *Mannheimia haemolytica* strain MB1401 | 4.00E-127 | 99% | KT013278.1 |
| FJ893232.1.1357 | 0.02851 | *Mannheimia* | *Mannheimia haemolytica* | 6.00E-130 | 100% | KT013278.1 |
| AY425293.1.1350 | 0.01488 | *Mannheimia* | *Mannheimia varigena* strain HSR GLF138 | 1.00E-126 | 99% | KM389532.1 |
| JQ454294.1.1395 | 0.01446 | *Mannheimia* | *Mannheimia varigena* strain HSR GLF138 | 6.00E-125 | 99% | KM389532.1 |
| New.ReferenceOTU53 | 0.00748 | *Mannheimia* | *Mannheimia varigena* strain HSR GLF138 | 1.00E-126 | 99% | KM389532.1 |
| EU826048.1.1357 | 0.00588 | *Mannheimia* | *Mannheimia varigena* strain HSR GLF138 | 2.00E-125 | 99% | KM389532.1 |
| AJ290754.1.1466 | 0.00547 | *Mannheimia* | *Mannheimia varigena* strain HSR GLF138 | 4.00E-127 | 99% | KM389532.1 |
| KF098486.1.1356 | 0.00522 | *Mannheimia* | *Mannheimia varigena* strain HSR GLF138 | 5.00E-126 | 99% | KM389532.1 |
| New.ReferenceOTU21 | 0.00497 | *Mannheimia* | *Mannheimia varigena* strain HSR GLF138 | 1.00E-106 | 95% | KM389532.1 |
| DQ272505.1.1555 | 0.00455 | *Mannheimia* | *Mannheimia varigena* strain HSR GLF138 | 5.00E-126 | 99% | KM389532.1 |
| GQ358869.1.1499 | 0.00301 | *Mannheimia* | *Mannheimia haemolytica* strain NIVEDI/MHS-4 16S ribosomal RNA gene, partial sequence | 6.00E-130 | 100% | KU598687.1 |
| GQ112346.1.1361 | 0.00276 | *Mannheimia* | *Mannheimia varigena* strain HSR GLF138 | 2.00E-125 | 99% | KM389532.1 |
| New.ReferenceOTU1077 | 0.00157 | *Mannheimia* | *Mannheimia haemolytica* strain MB1401 | 3.00E-123 | 99% | KT013278.1 |
| JQ454249.1.1402 | 0.00144 | *Mannheimia* | *Mannheimia varigena* strain HSR GLF138 | 1.00E-126 | 99% | KM389532.1 |
| HQ775782.1.1450 | 0.00135 | *Mannheimia* | *Mannheimia varigena* strain HSR GLF138 | 4.00E-127 | 99% | KM389532.1 |
| JQ448691.1.1371 | 0.00121 | *Mannheimia* | *Mannheimia haemolytica* strain MB1401 | 3.00E-128 | 99% | KT013278.1 |
| New.ReferenceOTU196 | 0.00102 | *Mannheimia* | *Mannheimia varigena* strain HSR GLF138 | 2.00E-100 | 93% | KM389532.1 |
| JQ448773.1.1397 | 0.00072 | *Mannheimia* | *Mannheimia haemolytica* strain MB1401 | 4.00E-127 | 99% | KT013278.1 |
| JQ449534.1.1394 | 0.00055 | *Mannheimia* | *Mannheimia varigena* strain HSR GLF138 | 4.00E-127 | 99% | KM389532.1 |
| New.ReferenceOTU6075 | 0.00055 | *Mannheimia* | Uncultured bacterium clone Ch_c77 16S ribosomal RNA gene, partial sequence | 3.00E-118 | 97% | EU995367.1 |
| New.ReferenceOTU1973 | 0.00044 | *Mannheimia* | *Mannheimia varigena* strain HSR GLF138, | 6.00E-115 | 96% | KM389532.1 |
| New.ReferenceOTU543 | 0.00041 | *Mannheimia* | *Mannheimia varigena* strain HSR GLF138 16S ribosomal | 3.00E-93 | 91% | KM389532.1 |
| New.ReferenceOTU1058 | 0.00041 | *Mannheimia* | *Mannheimia varigena* strain HSR GLF138 | 1.00E-116 | 97% | KM389532.1 |
| AY362918.1.1362 | 0.00036 | *Mannheimia* | *Mannheimia haemolytica* strain MB1401 | 3.00E-128 | 99% | KT013278.1 |
| New.ReferenceOTU986 | 0.00036 | *Mannheimia* | *Mannheimia varigena* strain HSR GLF138 | 3.00E-118 | 99% | KM389532.1 |
| New.ReferenceOTU6847 | 0.00028 | *Mannheimia* | *Mannheimia varigena* strain HSR GLF138 | 2.00E-95 | 92% | KM389532.1 |
| AJFQ01000001.75217.76732 | 6.1656 | *Mycoplasma* | *Mycoplasma bovirhinis strain: PG43* | 8.00E-129 | 100% | LC158834.1 |
| Y00149.1.1537 | 5.9036 | *Mycoplasma* | *Mycoplasma dispar* | 3.00E-128 | 100% | CP007229.1 |
| EF424082.1.1434 | 0.9125 | *Mycoplasma* | *Candidatus Mycoplasma haemobos* | 3.00E-128 | 100% | EF616467.1 |
| FJ226571.1.2338 | 0.1988 | *Mycoplasma* | Mycoplasma dispar | 3.00E-128 | 100% | CP007229.1 |
| X62699.1.1547 | 0.1259 | *Mycoplasma* | Mycoplasma dispar | 1.00E-127 | 99% | CP007229.1 |
